# Towards a typology of mental health crisis care services for children and young people in England and Wales: a cross-sectional survey and analysis of implementation practices

**DOI:** 10.1186/s12913-025-13446-6

**Published:** 2025-12-08

**Authors:** Clare Bennett, Claire Fraser, Ben Hannigan, Leanne Sawle, Jessy Williams, Aled Jones, Steven Pryjmachuk, Mair B. Elliott, Martin Elliott, Nicola Bennett, Euan Hails, Iain McMillan, Rachael Vaughan

**Affiliations:** 1 School of Healthcare Sciences, Cardiff University, Cardiff, CF14 4XN Wales, UK; 2 University Hospitals Birmingham, Birmingham, UK; 3 University of West London, London, UK; 4 School of Health Sciences, The University of Manchester and Manchester Academic Health Science Centre (MAHSC), Manchester, M13 9PL UK; 5 School of Geography, Earth and Environmental Sciences, University of Plymouth, Plymouth, UK; 6 School of Nursing and Midwifery, University of Plymouth, Devon, PL4 8AA UK; 7 Independent Consultant, Wales, UK; 8 CASCADE School of Social Sciences, Cardiff University, Cardiff, UK; 9 NHS Wales Aneurin Bevan University Health Board, Newport, NP18 3XJ Wales, UK; 10 Vale of Glamorgan Council, Barry Docks Office, Barry, CF634RT Wales

**Keywords:** Children and young people, Crisis care, Implementation, Mental health, Models, Normalisation process theory, Service mapping, Survey, Systems, Typology

## Abstract

**Background:**

The mental health and wellbeing of children and young people is a global concern. Alongside approaches emphasising mental health promotion in schools, communities and in the home, many countries are also investing in crisis services. These aim to meet the needs of young people experiencing acute psychosocial distress. A recent synthesis of the international evidence found a paucity of research in this area. This study sought to address this gap while simultaneously situating the findings within the international context and drawing out implications for policymakers and practitioners.

**Methods:**

A cross-sectional study aiming to describe and map approaches to the implementation and organisation of crisis care for children and young people was conducted across England and Wales. Complexity ideas, systems thinking and normalisation process theory conceptually underpinned the study. A bespoke survey captured service characteristics, service organisation and service user characteristics. It also incorporated the NoMAD tool to gather data on implementation. Usable data were received from 124 services. We used descriptive statistics and thematic analysis to summarise service characteristics and to develop a logic model. Typological analysis was used to develop a typology of service responses. NoMAD data were analysed using frequency analysis, item means and mean scale scores for each construct.

**Results:**

The ‘community in-person rapid response’ is the most common approach to provision. However, our analysis captured a patchwork of diverse provision across the system, typified by an absence of consensus regarding definitions of ‘mental health crisis’, lack of common agreement relating to the goals of care, and multiplication of approaches to the organisation and provision of services. Despite this, high levels of within-service coherence, cognitive participation and reflexive monitoring were observed.

**Conclusions:**

There is significant variation in the organisation and provision of crisis services for children and young people. Through situating our findings in a prevailing international policy context, we suggest that the variation we observe reflects an absence of a developed evidence base and a proliferation of strategies and frameworks which fail to provide clear guidance on how crisis care might best be organised and provided.

**Project registration:**

This project is registered with Research Registry (unique identifier: researchregistry8660).

**Supplementary Information:**

The online version contains supplementary material available at 10.1186/s12913-025-13446-6.

## Introduction

Despite the United Nations’ Sustainable Development Goals including Target 3.4, relating to the promotion of mental health and well-being [1], significant rates of mental health conditions, suicidality and self-harm among children and young people persist internationally [2–4]. Around the world, responses have included both upstream approaches (for example, through school-based activities intended to promote wellbeing) and downstream approaches (for example, through the provision of specialised services to young people experiencing mental health difficulties). As part of the downstream response to children and young people already in extreme psychosocial distress, which is our working definition of mental health crisis, a plethora of crisis care services have developed internationally [5].

Although policy reform is at the forefront of such changes [3, 6–9], our recent evidence synthesis identified a notable paucity of research into children and young people’s crisis care provision globally, but particularly in the UK [5]. Indeed, of the 58,000 citations identified, just three research studies originating from the UK met the review’s inclusion criteria [10–12]. From the limited UK research and non-research evidence it was identified that, in a crisis, choice, accessibility and support are important to children and young people and their families [10]. It was also identified that variability exists in the availability of services and hospital admissions across the UK [11]. Services for children and young people with learning disabilities or drug/alcohol issues were highlighted as particularly problematic with long waiting lists and many young people describing negative experiences of facilities and care [12]. In the UK literature it is acknowledged that children and young people often have difficulties accessing support from specialist child and adolescent mental health services (CAMHS) and/or primary care [13–15], with the emergency department (ED) being increasingly used for children and young people in crisis because of a lack of alternatives [15] and an absence of information for families outlining alternative support [16]. Although some young people report supportive and understanding staff in the ED [17], concerns about waiting times [18] and follow-up care after discharge from EDs are frequently raised [12, 13]. Some children and young people requiring crisis care in the UK report being ‘passed around’ when in crisis [16], with some ultimately taking their own lives [19].

Our underpinning evidence synthesis also identified that barriers to accessing UK services include difficulties securing care when the child or young person resides in a different locality to where the service is based [10, 13] and the age appropriateness of services when the young person is 16–17 years [16]. Although some areas have crisis services for children and young people, our review identified that availability in different areas is variable, with some services only operating within office hours [16]. UK case examples included in the review describe a number of different approaches to home-based crisis care [16, 20–22] with mental health local transformation plans in England including the development of intensive home treatment services [20].

More recent research into children and young people’s mental health crisis services has been conducted at the local level [23]. However, a lack of research investigating the current provision of mental health crisis services for children and young people across England and Wales remains. The work reported on in this article, which forms part of CAMH-Crisis2, a wider National Institute for Health and Care Research (NIHR) Health and Social Care Delivery Research programme-funded study [24, 25], aims to address this gap while simultaneously situating the findings within the international context and evidence base and drawing out implications for policymakers and mental health practitioners.

## Methods

### Aims

The specific research questions for this arm of the study were:

- What service models are currently in place in England and Wales to meet the needs of children and young people (up to the age of 25) in mental health crisis?
- What are the characteristics of these models?

Our overarching aim was to describe and map NHS, local authority, education, third sector and other approaches to the implementation and organisation of crisis care for children and young people across England and Wales. The objectives were to:

1. Co-create and widely disseminate a survey regarding service characteristics and implementation across all sectors that provide crisis services to children and young people in England and Wales.
2. Conduct descriptive statistical analysis of the survey results to inform the development of a typology of mental health crisis services for children and young people in England and Wales.
3. Situate the service map and emergent typology in the current body of international literature pertaining to children and young people’s mental health crisis services.

### Design

A cross-sectional approach was employed, drawing on established mapping exercise methods developed by Pryjmachuk et al. [26]. We began by developing a comprehensive database of services known to provide, or suspected to be providing, crisis care for children and young people, to facilitate subsequent survey distribution. Between November 2022 and January 2023, online search strategies were employed to identify services that described themselves as providing mental health crisis care for children and young people in health, social care, education, children’s services, youthwork, police and criminal justice settings, provided and commissioned by statutory, third sector and private operators across England and Wales. In addition, websites of known service providers and commissioning organisations (for example, NHS England, Local Health Boards in Wales, local authorities) were interrogated along with the Nexis database [27] and online service directories (for example, Youth Wellbeing Directory). Expert informants from the research team, study advisory group and independent project oversight committee were also consulted. Additionally, contacts for distribution lists (for example, managed through JISCMAIL) and professional/membership organisations were included in the database to support requests for the survey invitation to be distributed amongst members. Finally, we undertook Freedom of Information (FOI) requests to a range of Government departments and agencies to establish whether information was available on ‘current mental health crisis services for children and young people (up to 25 years) in England/Wales’.

Concurrently, a survey was developed. This was informed by the data collection instrument of our related study [26], our evidence synthesis [5] and consultation with young people (via CASCADE Voices, a young person’s research advisory group located in Cardiff, UK), clinicians and researchers in the field. The survey addressed: the characteristics of services (for example, setting, commissioning arrangements and history); organisation (for example, whether crisis services were standalone or part of an overall service specification, hours of operation, response times and staffing); service user characteristics (for example, age group served and numbers of young people seen); service delivery (for example, goals of services, referral routes and types of intervention provided); and evaluation and research (whether any previous evaluations had been conducted). In addition, to gather data on the implementation of services, statements derived from the NoMAD tool [28] were incorporated. NoMAD is a normalisation process theory-informed survey instrument created to improve understanding of complex interventions and their integration in health care contexts. It includes items addressing (amongst other areas) how normal an intervention feels to respondents, how it differs from usual ways of working and what training has been provided. NoMAD has been validated, is free to use, and can be adapted and incorporated pragmatically into project-specific data collection instruments [29, 30].

The survey was piloted and refined and, subsequently, an online version was created using JISC Online Surveys version 2 [31]. This version was piloted once again, further refined, with the final iteration being made available in both English (Supplementary File 1) and Welsh (Supplementary File 2). Hard copies in both languages were also made available. Survey invitations were distributed to all database contacts between January 2023 and February 2024. Additionally, professional networks and social media were used to raise awareness of the survey. Responses were continually mapped against postcode areas and service provider type, to monitor any specific gaps in survey returns. Accordingly, targeted desk-based internet searching was conducted to further develop the database of contacts and identified services were contacted by email or post. In total, we identified and contacted 1,484 (1,332 email and 152 postal) potential services.

### Data analysis

Descriptive analysis was employed to summarise data related to service characteristics and specifications [32] using Microsoft Excel Version 2023 [33]. Where data were missing, a default textual label of ‘missing data’ was employed; missing data were reported on but not included in the analyses. Where responses were not clear, the default textual label ‘unclear response’ was used; since this represented a response this was included in the analyses. Free text responses were examined using thematic analysis [34]. A logic model was developed throughout the data analysis phase of the study [35] to allow systematic consideration of the key components of the current ‘system’ of children and young people’s crisis care provision across England and Wales. By demonstrating the relationships between the collective resources, activities and intended outcomes of the various services included in this research, it was envisaged that we would be able to identify the underlying theory and assumptions of the system in its totality.

From the self-reported data, a typology of service responses was developed iteratively, using a typological analysis to aid categorisation [36, 37]. In addition, the process was informed by our evidence synthesis [5] and Dalton-Locke et al.’s [38] mapping of crisis resolution teams for adults and current healthcare policy. However, the rapidly changing landscape of crisis care provision for children and young people, particularly in relation to the increase in digital transformation during and since the Covid pandemic, limited the applicability of previous research to the current context. Evans et al.’s [5] and Dalton-Locke et al.’s [38] frameworks were, therefore, modified and developed to reflect the survey data through a process of constant comparison. As the typology emerged, the survey data were re-coded by two researchers (CF and LS) independently, specifically examining characteristics such as reported response times and the nature of the service provided, until inter-rater consensus was reached; when consensus could not be achieved, the wider research team was consulted. The typology was then presented to clinicians and researchers in the field for expert review until consensus was reached.

The NoMAD data [30] consisted of three 11-point and twenty 5-point Likert scales ranging from ‘strongly disagree’ to ‘strongly agree’. Responses were exported from Microsoft Excel, cleaned and imported into IBM SPSS Statistics Version 28.0 [39]. Data were analysed descriptively to produce a summary of how each service has been normalised, using frequency analysis data and item means. Following the guidance of the tool creators [28, 40], total NoMAD scores were not calculated. However, since internal consistency is established for the NOMAD tool [29], and consistent with the approach taken by Cook et al. [41], mean scale scores were calculated for each of NoMAD’s four constructs: coherence, cognitive participation, collective action and reflexive monitoring. One item from the collective action construct (‘crisis care for children and young people disrupts working relationships’) was reverse scored prior to calculating mean scale scores due to this being a negatively worded item (see Table 5).

Throughout, reporting has reflected the Checklist for Reporting of Survey Studies (CROSS) 4.

### Theoretical framework

Mental health crisis responses are complex interventions because they rest on the collective, purposeful, actions of multiple individuals interacting together in particular contexts [42]. A disparate range of organisations and individuals are tasked with the work of meeting children’s and young people’s needs when they are in crisis, their interactions taking place in a nested, or layered, environment [43]. ‘Context’ in this study was, therefore, key and we have paid close attention to the complexity of the system within which mental health crisis care, as a complex intervention, is introduced [44, 45]. Complexity theory and systems thinking therefore underpinned this aspect of the wider study [25], with the focus on understanding the rapidly changing nature of interventions and systems [46] which, in turn, give rise to dynamic and emergent behaviours [47–49]. In addition, normalisation process theory [50] informed our understandings of complex interventions and their integration in health care contexts, through application of the NoMAD tool [28].

### Reflexivity

As a research team, we actively employed reflexive strategies throughout the course of the study through peer discussion, supervision and engagement with expert groups. Although the research team included a carer and a young person mental health activist and research advisor, we were aware that we lacked cultural and socio-economic diversity. We were also substantially older than the children and young people we were focusing on and intended to include in the study. We, therefore, prioritised patient and public involvement in each aspect of this study by working with CASCADE Voices, a group of care-experienced young people (some of whom also have experience of using CAMHS and crisis services) advising on research.

### Ethical considerations

The Health Research Authority’s Decision Tool [51] determined that this aspect of the study did not meet the criteria for NHS ethics review. We, therefore, sought and secured a favourable ethics opinion from the School of Healthcare Sciences Research Ethics Committee in Cardiff University (REC983) as part of our commitment to good research practice [52]. Prior to survey completion, participants were provided with a copy of the participant information sheet, were given an opportunity to contact the research team to ask any questions, and completed an online consent form which followed the principles for ensuring valid, informed consent deemed appropriate by the Cardiff University School of Healthcare Sciences. Each participant was able to download a copy of their consent form. For participants wishing to complete a paper version of the survey, the same consent form was made available in paper format. Any data that were not accompanied by a valid consent form were excluded from analyses. All data has been stored securely in line with current University and NHS research governance and general data protection regulations.

## Results

### Findings

#### Survey responses

**Fig. 1:**
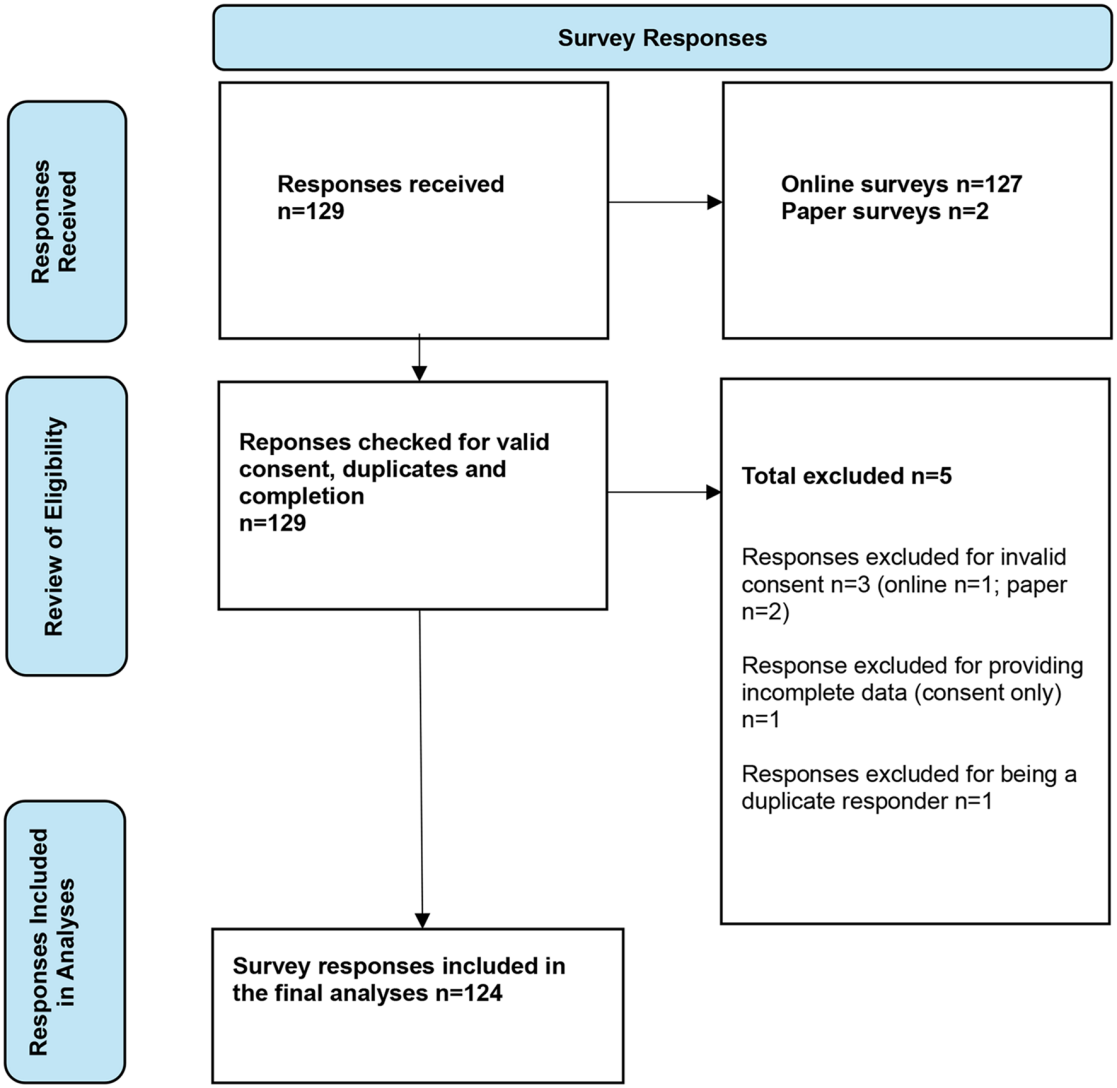
Flow diagram of survey responses from collection to inclusion in the data analyses

Figure 1 (adapted from the work of Page et al., 2021) presents the flow of survey responses from submission through to inclusion in the data analyses. Table 1 outlines the proportion of responses by region.

**Table 1:** Responses by region

Region	Frequency (*n*)	Percentage (%) of valid responses
North Wales	7	5.6
Mid and West Wales	4	3.2
South Wales West	6	4.8
South Wales East	4	3.2
South Wales Central	7	5.6
Across Wales	7	5.6
UK-wide/Multiple Regions	4	3.2
West Midlands	6	4.8
South East	14	11.3
Greater London	15	12.1
North West	17	13.7
South West	10	8.1
North East	3	2.4
East Midlands	3	2.4
East of England	6	4.8
Yorkshire & Humber	11	8.9
**Total number of valid responses**	**124**	**100**

Although all regions were represented, it is acknowledged that weighting was not employed, meaning that some regions may have been underrepresented.

#### Definitions of mental health crisis

In a free-text question on defining mental health crisis, a third of respondents (*n* = 42) either offered no definition or provided a service description. The remaining responses suggested a fluidity of definitions with the majority addressing a number of themes. Sixty-eight of the definitions referred to mental health crisis as a set of symptoms, such as a deterioration in daily functioning, self-harm, suicidal intent, extreme distress and destructive behaviours that are harmful to self or others. Fourteen described symptoms of crisis as ‘severe’ and 24 referred to crisis being ‘acute’, requiring an immediate response with the young person being at immediate or imminent risk. The need for support from others, for example mental health services, arose in eight of the responses. Fourteen participants described crisis as being self-defined by the young person. Twenty referred to a link with pre-existing mental health issues and three referred to wider causes such as socio-economic factors, waiting times, transitioning from CAMHS to adult mental health services and a lack of access to services for children and young people who are neurodivergent.

#### Service user characteristics

**Fig. 2:**
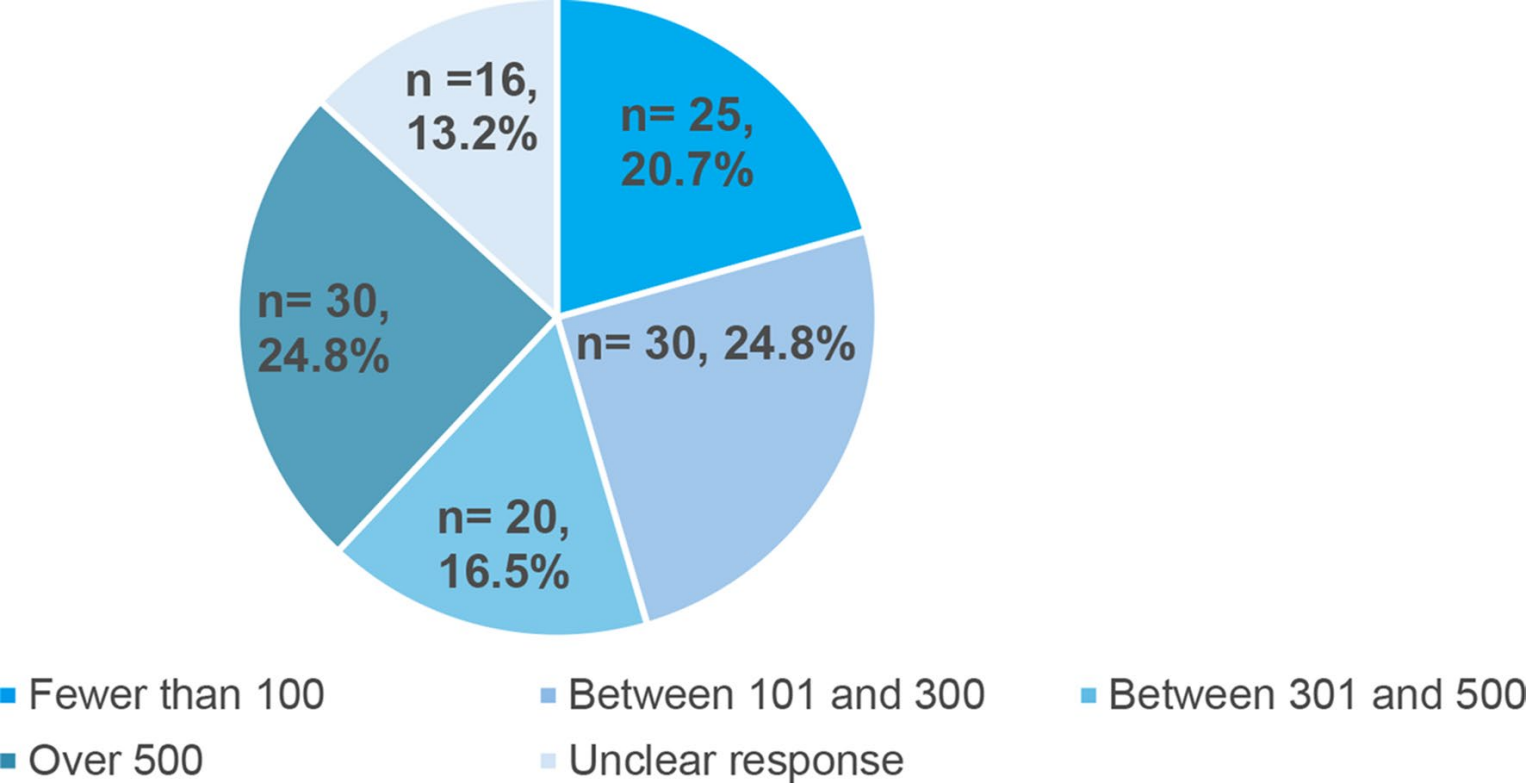
Number and percentage of children and young people using services in the preceding 12 months (*n* = 121)

Multiple responses were permitted to describe the age of service users. Ninety-six respondents (77%) indicated that they worked with 5–11 year olds, 121 (98%) worked with 12–18 year olds and 38 (31%) worked with young people aged over 18 years. Few services offered provision for specific groups: care leavers (*n* = 6), young people in the youth justice system (*n* = 6), those with disabilities (*n* = 2), children and young people who are neurodiverse (*n* = 2), those who had refugee status or were seeking asylum (*n* = 1), children and young people who had been affected by crime and/or abuse (*n* = 2), those with substance abuse problems (*n* = 1), and children and young people facing homelessness or at risk of family breakdown (*n* = 2). No other groups were described as being specifically targeted. Figure 2 provides a breakdown of service usage during the 12 months preceding survey completion. In response to a multiple response question about service users, 119 responses were received. All services reported working with children and young people; many also worked with family members and carers (*n* = 98, 82.4%) and third parties (*n* = 86, 72.3%), for example, friends or flatmates.

#### Service characteristics

The description of locales in which services operated often intersected, with multiple responses offered in the 123 responses received: rural (*n* = 55, 44.7%), semi-rural (*n* = 63, 51.2%), town (*n* = 83, 67.5%), city (*n* = 75, 61.0%), online (*n* = 29, 23.6%) and other (*n* = 6, 4.9%). Of the 124 responses received, 67 (54%) indicated that the service was a statutory mental health service (i.e. required by law and government funded), 23 (18.5%) were non-statutory (i.e. not required by law, may receive government funding but may be a charity or self-funded), 27 (21.8%) were statutory non-mental health services and seven (5.6%) were partnerships. The settings in which services were described as operating are presented in Table 2; ‘other’ included ambulance and mobile services.

**Table 2:** Setting(s) in which the service operates (* multiple answers permitted)

Service Setting (valid responses *n* = 124)	Provides service in setting (n*)	Percentage of valid responses providing service in setting (%)
A&E department	40	32.3%
Community health site	54	43.5%
Community non-health site	48	38.7%
Criminal justice setting	15	12.1%
Education (School)	26	21.0%
Education (Further Education/Sixth Form)	18	14.5%
Education (Higher Education)	10	8.1%
Education (Specialist setting)	19	15.3%
Education/NHS partnership	15	12.1%
Home setting	37	29.8%
Inpatient setting	27	21.8%
Remote (online, phone)	49	39.5%
Outpatient setting	26	21.0%
Youth group	15	12.1%
Other	10	8.1%

Of the 124 responses, the majority (*n* = 71, 57.7%) indicated that their particular service had been operational for over five years, 34 (27.6%) had been in operation for between one and five years and 13 (10.6%) had been operating for less than one year. Additional responses indicated a lack of knowledge of the operational history of the service. Thirty-four (28.8%) indicated that the service was bespoke and only provided crisis care, whereas the majority (*n* = 86, 71.2%) indicated that crisis care was provided as part of an overall service specification. Ninety-six (80%) of participants reported that their service was only for children and young people rather than open to all ages.

Services were accessed via various routes, with 123 respondents returning multiple responses. The majority (*n* = 110, 89.4%) indicated that professional referral was required, 70 (56.9%) indicated that their particular service could be accessed through self-referral and 58 (47.2%) indicated that referral from parents or carers was permitted. Two hundred and twenty-three criteria for access were identified from 115 free text survey responses; these were classified into five high-level themes (Table 3).

**Table 3:** Criteria for access (*n* = 115 responses)

Criteria	Sub-theme	*n*
Individual characteristics	*Age* *Neurodiversity* *Current service users/or has a key worker* *Education*,* Health and Care Needs Assessment (EHCNA) status* *Refugee status* *Drug/alcohol addiction (linked to mental health disorder)* *Not accessing a core service* *Abuse/trauma experience* *Registered with a GP and/or accessing CAMHS or another care team*	41 3 3 1 1 1 1 3 5
Open access	*Self-referral/crisis line route*	16
Risk factors	*Actual or thoughts of physical and/or mental harm to self (that could or has resulted in mental health unit admission*,* or hospital treatment)* *Harm to others* *Offending* *Exploitation* *Mental or physical harm from others (could be associated with a mental health disorder*,* or a crime/bullying)* *Escalating*,* complex and/or unstable presentations impairing function* *Impaired parental capacity/risk of family breakdown (due to mental health)* *Admission avoidance - reason unspecified* *Low level risk but intervention needed to prevent escalation*	60 9 2 2 2 12 3 5 1
Situational	*Universities* *Youth justice system* *Recurrent admissions or imminent*,* current or recent discharge from in-patient MH unit* *Section 136 suite* *Hospital e.g. Paediatric ward/A&E/acute hospital/Single point of access (SPA) or CAMHS triage* *Looked-after children (LAC)/edge of care* *School refusal* *Schools* *Geographical locale*	4 3 5 2 11 6 1 1 13
Professional referral and/or collaborative working	*Referral and/or collaboration with a GP*,* or other service provider*	6
**Total number of criteria**		**223**

**Fig. 3:**
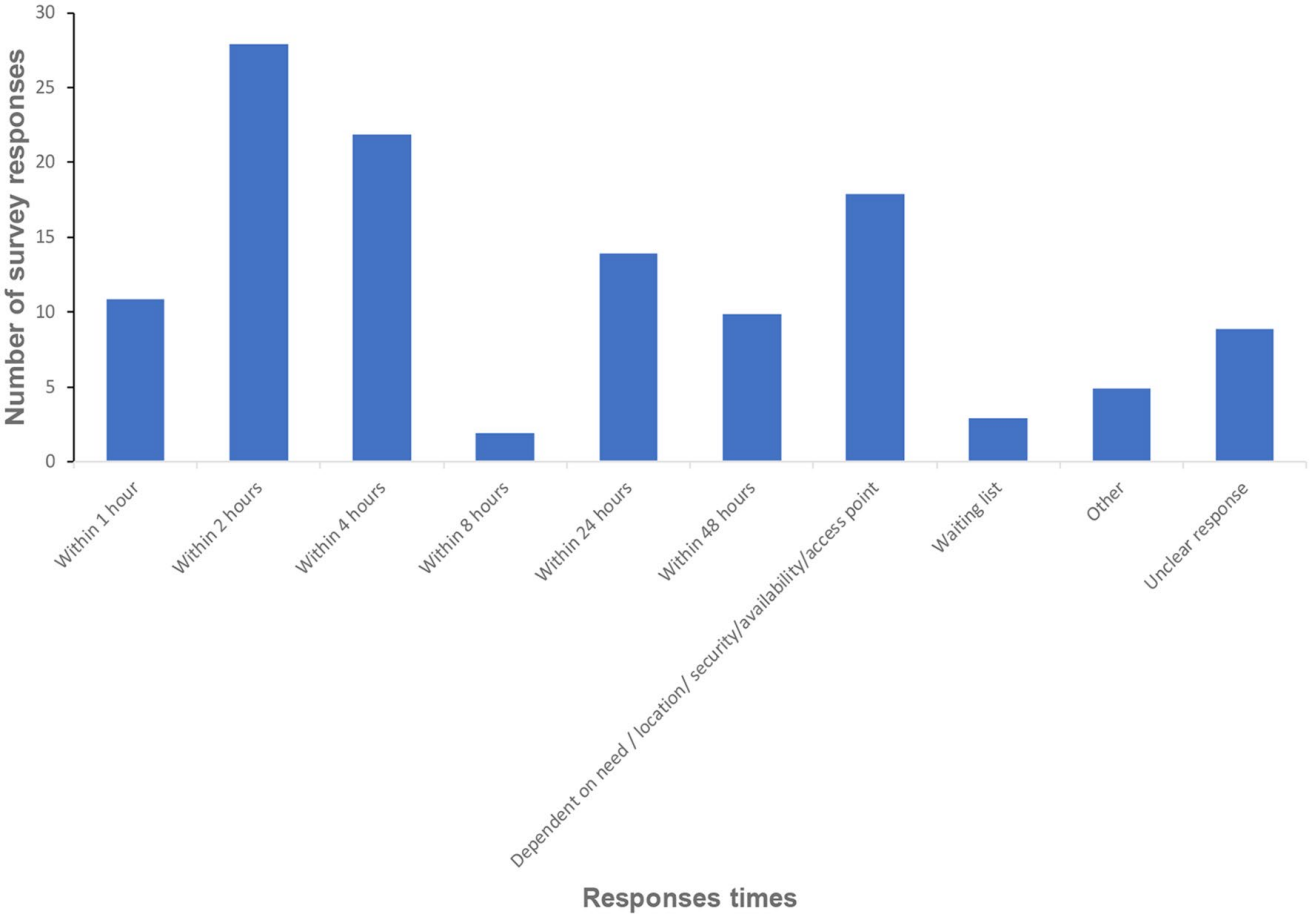
Response times of services (*n* = 122 responses)

Figure 3 outlines data from a multiple answer question about response times. Respondents described a range of response times, varying from within one hour (*n* = 11, 9%) to within 48 h (*n* = 10, 8.2%).

#### Therapeutic modalities

**Fig. 4:**
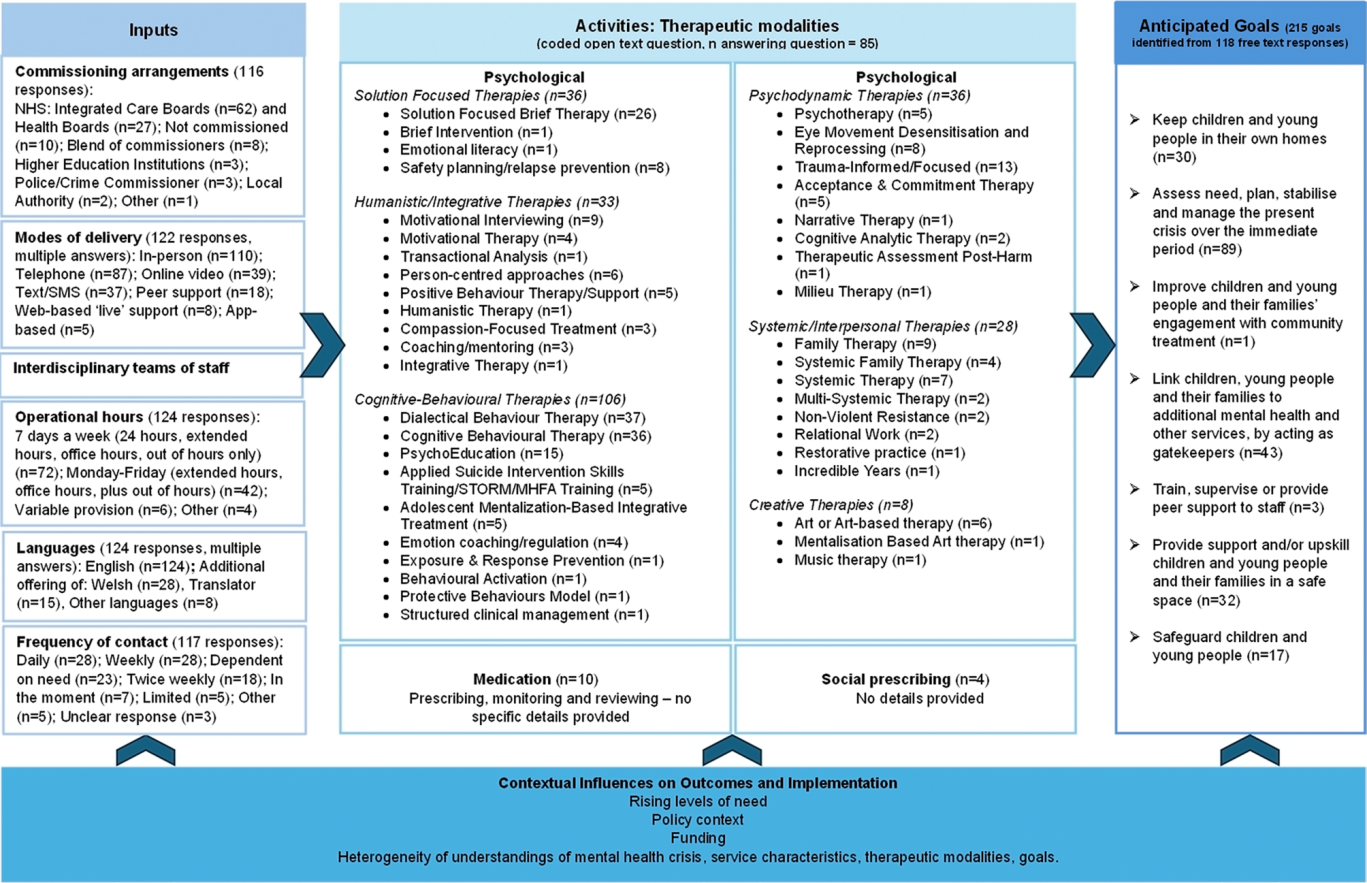
Logic model detailing children and young people’s crisis interventions and intended outcomes

Figure 4 provides a logic model of children and young people’s crisis interventions and intended outcomes. It summarises a range of resources, or inputs, in the form of commissioning arrangements, modes of service delivery, interdisciplinary teams of staff, the operational hours of services and the languages available in the services described. The activities focus on the therapeutic modalities available across the services; from the 124 survey responses received, eligible data suitable for analysis of therapeutic modalities was provided in 85 of the survey responses. Analysis revealed a range of biopsychosocial approaches [53] to therapeutic intervention. Whilst biological (medication) and social (social prescribing) interventions were cited by only a small number of respondents, all but one of the 85 survey respondents described at least one psychological therapeutic intervention that was provided for children and young people (and sometimes family members) accessing the service. Many respondents described a range of different interventions available to service users indicating that a choice of therapeutic modalities is often available. Indeed across the 85 survey responses, 247 different psychological interventions were described, indicating a remarkable heterogeneity in the range of therapeutic modalities employed across crisis care responses. A summary of therapeutic modalities is provided in Fig. 4, along with a summary of anticipated goals.

#### Typology

Each of the services within the survey responses were categorised by the research team into one of 15 distinct models, or types (Table 4). The table provides a summary of codes, model titles, definitions, mode of provision, intervention type, setting and the number of services that led to the development of the particular category. Of the 124 services, the majority were community in-person responses (*n* = 42) and a blend of community in-person and hospital-based rapid responses (*n* = 31). The remaining 13 models comprised lower frequencies of services, ranging from *n* = 1 to *n* = 13.

**Table 4:** Typology of children and young people’s mental health crisis services in England and Wales (*n* = 124)

Code	Model Title	Definition	Mode of provision	Intervention type	Setting	*n*
A	Community in-person rapid response	Primarily an in-person response, which provides rapid mental health crisis assessment, triage and/or support for children and young people in their community setting (e.g. education, youth justice, home, community, or other non-clinical setting). May involve multiple partners.	Primarily in-person	Assessment, triage and/or support	Community	42
B	Emergency mobile response	An emergency mobile response where first responders provide crisis assessment, triage and/or support for children and young people in mental health crisis (e.g. police, ambulance, joint responses with mental health staff) as needed.	In-person	Assessment, triage and/or support	Community	3
C	Non-residential safe haven	A community-based service which provides assessment and immediate support for children and young people in mental health crisis in a given location (e.g. hub, one-stop shop, crisis café, drop-in). May involve NHS and third sector.	Primarily in-person	Assessment and support	Community	8
D	Remote provision	A service which primarily provides remote crisis assessment, triage and/or support via the use of technology to children and young people (e.g. telephone, text, video conference, apps).	Remote	Assessment, triage and/or support	Community	9
E	Day care	A non-residential day service providing support, activity and therapeutic groups, for children and young people in mental health crisis (e.g. community-based services, therapeutic day hospital services).	In-person	Assessment, triage and/or support	Community	1
F	Hospital-based rapid response	A hospital-based rapid response (e.g. accident and emergency department, psychiatric/clinical decision unit, outpatient clinic, inpatient mental health ward, paediatric ward), where assessment can be conducted and support plans developed for children and young people in mental health crisis. May involve residential stay.	In-person	Assessment, triage and/or support	Hospital	13
G	Residential crisis house	A residential service providing time-limited, non-hospital support for children and young people in mental health crisis.	In-person	Assessment and support	Community residential	3
H	Assessment and/or triage only	Responses which are limited to assessment and/or triage and/or signposting onwards, but which do not extend to the provision of ongoing support.	In-person or remote	Assessment and/or triage only	Community or hospital	3
I	Community in-person and hospital-based rapid response with non-residential safe haven	Primarily an in-person response, which provides rapid mental health crisis assessment, triage and/or support for children and young people in their community setting, a hospital-based rapid response where assessment can be conducted and support plans developed for children and young people in mental health crisis and a community-based service which provides assessment and immediate support for children and young people in mental health crisis in a given location. May involve multiple partners including NHS and third sector.	Primarily in-person	Assessment, triage and/or support	Community and hospital	1
J	Rapid in-person community-based response, remote response and hospital-based rapid response	An in-person response providing rapid mental health crisis assessment, triage and/or support for children and young people in their community setting, remote crisis assessment, triage and/or support via the use of technology and a hospital-based rapid response where assessment can be conducted and support plans developed. May involve multiple partners and involve residential stay.	In-person and remote	Assessment, triage and/or support	Community and hospital	4
K	Community in-person and hospital-based rapid response	Primarily an in-person response, which provides rapid mental health crisis assessment, triage and/or support for children and young people in their community setting and a hospital-based rapid response where assessment can be conducted and support plans developed. May involve multiple partners and involve residential stay.	Primarily in-person	Assessment, triage and/or support	Community and hospital	31
L	Community in-person rapid response and crisis placements service	Primarily an in-person response, which provides rapid mental health crisis assessment, triage and/or support for children and young people in their community setting as well as offering short-term crisis foster placements, supported by local crisis services. May involve multiple partners.	Primarily in-person	Assessment, triage and/or support	Community and community residential	1
M	Community in-person rapid response and assessment and/or triage	Primarily an in-person response, which provides rapid mental health crisis assessment, triage and/or support for children and young people in their community setting as well as responses which are limited to assessment and/or triage and/or signposting onwards, but which do not extend to the provision of ongoing support. May involve multiple partners.	In-person and remote	Assessment, triage and/or support	Community or hospital	1
N	Non-residential safe haven and digital provision	A community-based service which provides assessment and immediate support for children and young people in mental health crisis in a given location as well as providing remote crisis assessment, triage and/or support via the use of technology. May involve NHS and third sector.	In-person and remote	Assessment, triage and/or support	Community	1
O	Digital provision and assessment and/or triage	Primarily provides remote crisis assessment, triage and/or support via the use of technology as well as responses which are limited to assessment and/or triage and/or signposting onwards, but which do not extend to the provision of ongoing support.	In-person or remote	Assessment, triage and/or support	Community or hospital	3

Using the service models as units of analysis, heterogeneity in response times was identified. For example, community in-person rapid response services’ (model A) response times ranged from less than one hour to within 48 h. Similarly, within model K, community in-person and hospital-based rapid response, response times varied from within two hours to within 48 h and services that were characterised as model C, non-residential safe havens, ranged from responding in less than one hour to within 48 h. Variability in operational hours was also a feature of all service models. For example, within model A, community in-person rapid response services, 47.6% of the 42 services were available within standard office hours while other services were available seven days a week with varying opening hours (24 h daily, office hours and extended hours) or extended office hours. Analysis of professional groups working across the models identified that social workers were the most widely represented. They were recorded in all but two models; E (day care) and N (non-residential safe haven and digital provision). Counsellors/therapists featured in all but three of the models; E (day care), H (assessment and/or triage only) and, O (digital provision and assessment and/or triage). Model A, community in-person rapid response services, and model F, hospital-based rapid response, reflected a diverse range of staff working in these services.

#### NoMAD

The original NoMAD has a Cronbach’s alpha coefficient of 0.89 indicating good internal consistency (Finch et al., 2018). In our study, Cronbach’s alpha was also 0.89 for the overall scale, indicating good internal consistency with our specific sample of crisis managers and practitioners. For context, alpha values between 0.7 and 0.8 are deemed satisfactory and ≥ 0.8 good. Cronbach’s alpha coefficients ≥ 0.7 are recommended for group comparison [54]. One hundred and fifteen respondents (93%) completed the NoMAD section of the survey. However, four provided insufficient data to be included in the analyses. Analyses presented here are, therefore, based on 111 responses. Of the 111 respondents, 73% (*n* = 81) were involved in managing or overseeing crisis care for children and young people and 27% (*n* = 30) were practitioners involved in delivering this care for children and young people in crisis.

Respondents were initially invited to respond to three items which Rapley et al. (2018) refer to as ‘global normalisation items’, focusing on whether providing crisis care feels familiar and a normal part of work, which are rated using a response scale ranging from 0 to 10 where 0 = not at all, 5 = somewhat and 10 = completely. Respondents’ answers (*n* = 108) to the global normalisation item, ‘When you provide crisis care for children and young people, how familiar does it feel?’ ranged from ‘feels completely familiar’ (*n* = 35, 32.4%) to ‘still feels very new’ (*n* = 2, 1.9%) with a mean rating of 8.83 (SD = 2.366) indicating a reasonably high level of familiarity. Respondents’ answers (*n* = 109) to the global normalisation item, ‘Do you feel that providing crisis care for children and young people is currently a normal part of your work?’, ranged from ‘completely’ (*n* = 48, 44%) to ‘not at all’ (*n* = 2, 1.8%) with a mean rating of 9.09 (SD = 2.355) indicating a high level of normalisation. Respondents answering 7 or below to this question were invited to answer a further question about whether normalisation was likely to occur in the future. Twenty-eight participants provided a response, with the majority (85%) indicating they felt it would, though this ranged from ‘somewhat’ likely to occur through to ‘completely’ likely to occur with a mean rating of 6.75 (SD = 2.50).

The remaining 20 items reflected the four original Normalisation Process Theory (NPT) core constructs, using a five-point Likert scale for each item. Ranking ranged from strongly disagree (score = 1) to neutral (score = 3) to strongly agree (score = 5). Table 5 presents frequencies to indicate where participants provided more positive or negative responses, as suggested by Finch et al. (2015), with not applicable responses removed. Item responses were additionally mapped onto the four theoretical NPT constructs to provide an overview of factors likely to affect normalisation of crisis services (Rapley et al., 2018). Participants were able to choose not to respond to individual statements if they did not feel it was relevant to their role, relevant at this stage or relevant to the intervention.

**Table 5:** Normalisation process theory frequencies of agreement, total scores and reliability coefficients

	Item (valid *n*)	Mean	SD	Agree *n* (%)	Neutral *n* (%)	Disagree *n* (%)
Coherence	I can see how crisis care for children and young people differs from usual ways of working (*n* = 108)	4.31	0.90	97 (89.8%)	5 (4.6%)	6 (5.6%)
	Staff in this service have a shared understanding of the purpose of crisis care for children and young people (*n* = 110)	4.23	0.92	95 (86.4%)	7 (6.4%)	8 (7.3%)
	I understand how crisis care for children and young people affects the nature of my own work (*n* = 108)	4.40	0.71	101 (93.5%)	5 (4.6%)	2 (1.9%)
	I can see the potential value of crisis care for children and young people for my work (*n* = 107)	4.60	0.66	102 (95.3%)	4 (3.7%)	1 (0.9%)
Cognitive Participation	There are key people who drive crisis care for children and young people forward and get others involved (*n* = 106)	4.11	0.89	85 (80.2%)	15 (14.2%)	6 (5.7%)
	I believe that participating in crisis care for children and young people is a legitimate part of my role (*n* = 105)	4.50	0.75	99 (94.3%)	1 (1%)	5 (4.8%)
	I’m open to working with colleagues in new ways to use crisis care for children and young people (*n* = 109)	4.70	0.48	108 (99.1%)	1 (0.9%)	0 (0.0%)
	I will continue to support crisis care for children and young people (*n* = 107)	4.69	0.54	105 (98.1%)	1 (0.9%)	1 (0.9%)
Collective Action	I can easily integrate crisis care for children and young people into my existing work (*n* = 104)	4.12	0.99	80 (76.9%)	15 (14.4%)	9 (8.7%)
	Crisis care for children and young people disrupts working relationships* (*n* = 97)	2.30	1.25	21 (21.6%)	10 (10.3%)	66 (68%)
	I have confidence in other people’s ability to use crisis care for children and young people (*n* = 105)	3.74	0.93	68 (64.8%)	29 (27.6%)	8 (7.6%)
	Work is assigned to those with skills appropriate to crisis care for children and young people (*n* = 102)	3.83	1.07	77 (75.5%)	11 (10.8%)	14 (13.7%)
	Sufficient training is provided to enable staff to implement crisis care for children and young people (*n* = 107)	3.58	1.12	64 (59.8%)	21 (19.6%)	22 (20.6%)
	Sufficient resources are available to support crisis care for children and young people (*n* = 107)	2.80	1.31	36 (33.6%)	21 (19.6%)	50 (46.7%)
	Management adequately supports crisis care for children and young people (*n* = 107)	3.92	1.05	80 (74.8%)	16 (15%)	11 (10.3%)
Reflexive Monitoring	I am aware of reports about the effects of crisis care for children and young people (*n* = 106)	3.83	1.02	77 (72.6%)	14 (13.2%)	15 (14.2%)
	The staff agree that crisis care for children and young people is worthwhile (*n* = 108)	4.52	0.69	101 (93.5%)	6 (5.6%)	1 (0.9%)
	I value the effects that crisis care for children and young people has had on my work (*n* = 107)	4.31	0.95	92 (86%)	9 (8.4%)	6 (5.6%)
	Feedback about crisis care for children and young people can be used to improve it in the future (*n* = 109)	4.62	0.69	106 (97.2%)	1 (0.9%)	2 (1.8%)
	I can modify how I work with crisis care for children and young people (*n* = 105)	4.38	0.80	93 (88.6%)	9 (8.6%)	3 (2.9%)

Construct Score Summaries		Mean	SD	95% CI	Range	Alpha
Coherence		4.41	0.52	4.31–4.51	3.00–5.00	0.73
Cognitive Participation		4.50	0.53	4.40–4.60	2.75–5.00	0.80
Collective Action		3.67**	0.72	3.53–3.80	1.29–5.00	0.80
Reflexive Monitoring		4.34	0.63	4.22–4.45	1.60–5.00	0.70

Key: *Negatively worded item ** Negatively worded item reverse scored prior to mean calculation

‘Coherence’ or the ‘sense-making work’ (Finch et al., 2015) that takes place in healthcare settings in order to implement an intervention into practice, involves understanding and planning at both the individual and collective level. In the current sample, particularly high levels of coherence were observed with 86–95% of respondents agreeing or strongly agreeing with each of the individual statements and a mean ranking of 4.41 (SD = 0.52) across the five-point Likert scales.

‘Cognitive participation’ describes the ways in which people work together to develop and sustain collective practice around an intervention. Three of the four cognitive participation items scored highly with over 90% of respondents agreeing or strongly agreeing with the items presented. Whilst a lower score overall, the majority (80%) also agreed or strongly agreed ‘there are key people who drive crisis care forward’. Overall mean for the four items was 4.50 (SD = 0.53).

‘Collective action’ describes the way people work together to operationalise an intervention. Levels of agreement with the collective action items were overall lower than those observed for the first two constructs. Only a third of respondents (33.6%) agreed or strongly agreed that sufficient resources were available to support crisis care and fewer than two-thirds (59.8%) agreed or strongly agreed that sufficient training was provided to enable staff to implement crisis care. This was reflected in a lower mean scale score of 3.67 (SD = 0.72).

‘Reflexive monitoring’ refers to the way that people reflect on and appraise interventions to understand their impact. Respondents indicated high levels of agreement with statements measuring reflexive monitoring on the NoMAD tool with 86–97% agreeing or strongly agreeing with four of the five items. A slightly lower level of agreement (72.6%) was observed for ‘I am aware of reports about the effects of crisis care’. The mean scale score for this construct was 4.34 (SD = 0.63).

Construct mean scores were highest in relation to the construct ‘cognitive participation’ (M = 4.50, SD = 0.53). Cronbach’s alpha ranged from 0.7 to 0.8 across the four NPT constructs, suggesting a good level of internal consistency.

To explore differences between sub-groups of respondents, mean scale scores were calculated for each construct (Table 6). Overall results suggest very little difference between the sub-groups and, indeed, statistical analyses (independent samples t-test and one-way Anova) found no statistically significant differences. However, the data suggests a pattern of marginally more positive responses among service managers compared to practitioners and, with the exception of ‘collective action’, bespoke crisis services compared to those that are part of an overall service specification. Likewise, a service history of one to five years appeared to result in slightly more positive responses relating to coherence, cognitive participation and reflexive monitoring, but this was not uniform, with services established over five years having the most positive mean scale construct score for ‘collective action’. Cross-sector partnerships consistently scored lower across the four constructs when compared with statutory and non-statutory services.

**Table 6:** Mean scale construct scores for respondent/service sub-groups

	Coherence		Cognitive Participation		Collective Action		Reflexive Monitoring	
	Mean	SD	Mean	SD	Mean	SD	Mean	SD
**Respondent Type**								
Service Manager (*n* = 80)	4.47	0.51	4.56	0.51	3.82	0.68	4.41	0.57
Practitioner (*n* = 30)	4.25	0.53	4.34	0.58	3.25	0.67	4.13	0.72
**Focus of Service**								
Bespoke Crisis Care service (*n* = 31)	4.54	0.48	4.60	0.46	3.62	0.78	4.43	0.53
Crisis care part of overall specification (*n* = 79)	4.36	0.53	4.46	0.56	3.68	0.70	4.30	0.66
**Service History**								
Less than 1 year (*n* = 13)	4.44	0.59	4.40	0.47	3.69	0.67	4.30	0.87
1–5 years (*n* = 30)	4.52	0.47	4.59	0.57	3.65	0.75	4.50	0.45
Over 5 years (*n* = 62)	4.38	0.52	4.52	1.49	3.74	0.67	4.31	0.61
**Service Sector**								
Statutory (*n* = 80)	4.40	0.53	4.58	0.46	3.67	0.71	4.34	0.67
Non-Statutory (*n* = 24)	4.46	0.51	4.29	0.67	3.71	0.83	4.30	0.55
Cross-sector Partnership (*n* = 6)	4.33	0.52	4.33	0.66	3.48	0.30	4.38	0.36

## Discussion

Far less is known about the organisation and provision of mental health crisis care for children and young people than is known about crisis care for adults, with services for adults in the UK being both longer established following the appearance of key policy drivers at the turn of the century [55] and better researched [38, 56–58]. Models of acute and crisis care for adults span a variety of approaches and settings [59], and approaches to provision for children and young people might fruitfully learn from these. In this current study, whilst acknowledging that our findings are limited by the number of responses, our analysis of survey data relating to the characteristics of crisis services for children and young people in England and Wales and the emergent logic model presented in Fig. 4 produce a number of important findings with relevance for policy and future provision. Table 4 above demonstrates the ‘community in-person rapid response’ as the most prevalent approach to provision, broadly reflecting NHS England [60, 61] and Welsh Government strategy [62]. Overwhelmingly, however, our analysis of included services reveals a patchwork system as a whole, typified by an absence of consensus regarding definitions of ‘mental health crisis’, a lack of common agreement relating to the goals of care, and a diversity of approaches to the organisation and provision of services. Our findings point to a remarkable heterogeneity of commissioning arrangements, settings, routes to access, periods of operation, response times and therapeutic modalities across services both within, and between, the models identified in our typology. The plethora of activities and interventions described across services, and the wide-reaching nature of their anticipated goals (which range from assessment, planning, stabilisation and management of crisis to staff training, supervision and peer support), is particularly striking. The risks of a patchwork of this type include that certain groups, with certain needs, such as those who identify as lesbian, gay, bisexual or transgender (LGBTQ) [63] and young people of minority ethnic groups [64] miss out on support, which could further exacerbate barriers to access and associated health inequalities. Variation also raises questions regarding the overall coherence of commissioning and of service development. Yet, despite this variation, data from the NoMAD component of our survey relating uniquely to the implementation of individual services also point to high levels of within-service coherence, cognitive participation and reflexive monitoring. Whilst across the system as a whole crisis care varies suggesting a lack of coherence at a macro-level, for key respondents within each service, local arrangements make sense, are collectively driven forward and are actively appraised.

The purpose of employing a logic model was to support the identification of an underlying theory and set of assumptions within the system in its totality, but this task has been challenged by the lack of agreement regarding the cross-sector aims of provision in a context in which policy, standards and guidance have multiplied but also lack clarity. In our foundational evidence synthesis [5] we drew attention to the range and diversity of reports, statements and related policy outputs produced by UK government, professional and other bodies with relevance to mental health care for children and young people. Yet, both the World Health Organization [65] and the NHS Confederation’s Mental Health Network [66] have recently highlighted a lack of investment in children and young people’s mental health, an observation which sits at odds with the policy thicket [67] surrounding this field. Over the last decade, England has seen the publication of a significant quantity of policy documents with a bearing on mental health provision for children and young people. These include: the Care Quality Commission’s Right Here, Right Now report [68], the Crisis Care Concordat [7], Future in Mind [6], the Five Year Forward View for Mental Health [8], the 5-year NHS Mental Health Implementation Plan (MHIP) [60], the NHS Long Term Plan [69] and national implementation guidance for urgent and emergency mental health care for children and young people [61]. Likewise, in Wales, children and young people’s mental health has been prioritised through a series of policies and governmental reports, including The Children and Young People’s Plan [70], No Wrong Door: Bringing Services Together to Meet Children’s Needs [71], Wellbeing of Wales, 2022: Children and Young People’s Wellbeing [72], the Framework on Embedding a Whole-School Approach to Emotional and Mental well-being [73] and a new all-age national mental health strategy [62], among others. This proliferation of policies reflects an urgency surrounding children and young people’s mental health which is felt both nationally and internationally: Canada, for example, is currently investing in co-designed mental health system transformation for children and young people [74] and the Australian government has recently launched a first National Children’s Mental Health and Wellbeing Strategy [75]. This ‘initiativitis’ may also reflect the politics of policy development, characterised by the existence of competing ideas of what it is which needs to be prioritised in a context of limited resources and an underdeveloped evidence base in which policymakers struggle to tackle the ‘wicked problem’ of mental ill-health in ways which demand both upstream and downstream responses [76].

In the UK, despite the production of detailed data on young people’s mental health [77] alongside the publication of multiple documents with relevance to provision we also note the lack of definitive guidance, advice or quality standards relating to crisis care from England and Wales’ National Institute for Health and Care Excellence. Current national mental health implementation guidance in England includes high-level expectations that crisis care for children and young people incorporate round-the-clock assessment, brief response and intensive home treatment [60] and further recent guidance for children and young people specifically sets out core crisis functions [61]. In Wales, the aspiration in a new all-age mental health and wellbeing strategy is for ‘appropriate and tailored’ crisis care for children and young people [62]. As guides for current and future provision both nations’ policy frameworks are sufficiently broad to support the kind of diversity in approaches which we have described in this paper. Variation in service types and ways of working may, therefore, reflect a lack of clear evidence of what works and the emergence of local responses to local needs in a national policy context which lacks absolute specificity over how ‘crises’ might be defined and assessed, what interventions might be offered, and how support overall might best be organised. Shaping the heterogeneity we have described will be the diverse geographies and locales in which services have emerged, with their contrasts in population need and histories of interagency arrangements and approaches to the organisation of care. In this sense, our finding of variation in the goals and provision of crisis care for children and young people reflects patterns previously described in the international literature in this field [5, 78, 79]. However, rather than offering reassurance, such patterns raise questions concerning capacity and sustainability and in the context of the workforce, the diverse staffing models employed and the wide range of interventions on offer highlight potential issues for education and training [80].

Whilst a lack of uniformity of service provision may lead to challenges for children, young people and their families in knowing what services are available in their particular locality and how to access them [23], at the micro-level of interaction between children, young people, their families and professionals, intervention variation may also have advantages. Indeed, our colleagues [81] have recently highlighted that a choice of therapeutic support is valued by children and young people accessing services. Additionally, the National Children’s Bureau [82] has emphasised the need for personalisation of services and therapies for children and young people requiring mental health interventions. What our data are not able to demonstrate, however, is the extent to which the heterogeneity we have observed in approaches to organisation and provision reflects the needs experienced, or the wishes expressed, by young people in each locale and how much it reflects system-level factors such as resource availabilities and commissioner, managerial and/or professional preferences. In this regard, whilst survey respondents indicated high levels of agreement with NoMAD statements relating to reflexive monitoring, this being the ways in which interventions are reflected on and appraised, as we noted above a lower proportion agreed with the individual item, ‘I am aware of reports about the effects of crisis care’. Space therefore exists to better evaluate the impact that different approaches to support exert on children, young people and their families and, in particular, those from vulnerable populations.

We anticipated that heterogeneity in provision may pose challenges for implementation. However, whilst cognisant that NoMAD data were limited by small and unevenly distributed sub-group samples (Table 6) and that responses represented the perspective of just one person, who was likely to be a manager (*n* = 80, practitioners *n* = 30) within each service, the pattern of responses at both the construct and item level suggest a consistently high level of implementation and normalisation, with the exception of ‘collective action’ (which refers to the work which people do, together, to realise a new intervention or service). Interpretations are limited by the nature of survey data, but we propose that these findings may suggest a high degree of local system readiness, or preparedness, to successfully adopt and integrate new practices underpinned by the sense of urgency conveyed by the significant volume of national and international policy and data in this space [83–85]. The lower level of agreement over collective action may reflect the current policy thicket in this field, which we found in our previous evidence synthesis [5], allied to the lack of specificity in current and emergent policy around what crisis care to provide and how. As our data demonstrate, multiple different innovations are emerging rapidly and simultaneously, against a backdrop of perceived limited resources and training. The absence or only partial presence of national standards, resources and training are highly consequential for local implementation; whilst they may not derail implementation completely, they can explain why implementation is patchy or partial.

Implicit in our analysis is a concern regarding the pace at which policymakers have unleashed a surfeit of downwards-directed actions in their zest to secure improvement in a field that is uniquely distressing for children and young people, their families, professionals and societies internationally. Whilst the prioritisation of children and young people’s mental health crisis policy is welcome, we urge caution in continuing the current proliferation in a context in which the evidence is limited and very little is known about the effects of change. Whilst not advocating for a ‘hands off’ approach, we would instead argue for a period of reflection and international collaboration in examining the most appropriate whole-systems approach to serve this growing population and ensure effective implementation. As has been observed in related studies in the children and young people’s mental health field [86], a balance in future service provision may need to be struck between the development and imposition of broad, evidence-informed, national standards and local flexibility in how these are assured in particular contexts.

### Limitations

Although our database of children and young people’s crisis services was extensive, spanning health, social care, education, children’s services, youthwork, police and criminal justice crisis services provided and commissioned by statutory, third sector and private operators, we doubt we have a comprehensive picture of the range of English and Welsh services available due to the limited response to survey invites. Despite using expert informants and social media to identify services and promote the study, as Pryjmachuk et al. [26] have identified, the largely online search strategies that we used restricted the search to services with an established online presence, potentially missing smaller services or statutory services provided by the NHS, local authorities or schools, that may be less likely to have an established online presence. Although we used the Nexis database [27] to identify such services and posted hard copies of the survey to those for whom we could not locate an email address, omissions were inevitable. Searches were also time-restricted and the study was cross-sectional, meaning that evolving services may have been missed. Compounding this, the total number of responses received was disappointing and, within responses, participants sometimes failed to respond to various aspects of the survey. There appeared to be no particular pattern to this, although a low number of responses to the negatively worded item in the NoMAD element of the survey (Table 5) was notable. Reflecting these limitations, the typology may also fail to reflect the true range of service models currently in operation across the two nations and should be seen as an evolving model. In addition, potential sampling bias may have skewed our findings and negatively impacted generalisability and equity.

## Conclusions

In this paper we have identified a patchwork of approaches to meeting children and young people’s mental health crisis needs in England and Wales and provided positive and negative interpretations of these approaches. Significant variation in organisation and provision is found within, and between, services grouped in our 15 distinct typologies. Implementation data suggest that, despite this heterogeneity, at local level services are coherent, and driven by people collaborating together and are (for the most part) appraised. Through situating our findings in a prevailing policy context we suggest that the variation we observe reflects a proliferation of strategies and frameworks which, whilst amounting to a complex thicket, neither draw on a mature evidence base nor provide clear guidance on how crisis services might best be organised and provided. One outstanding research task is to seek richer, more detailed, data on models of provision and on the experiences of young people and their families to inform national and international health policy. The next stage of this research project addresses this need by examining the delivery and experience of eight contrasting mental health crisis services for children and young people in England and Wales (paper under review).

## Supplementary Information

Below is the link to the electronic supplementary material.

Supplementary Material 1

Supplementary Material 2

Supplementary Material 3

## Data Availability

The data generated and analysed during the current study are not publicly available to protect the confidentiality and anonymity of participants. Survey responses could identify participants and individual services and cannot be made publicly available in accordance with research ethics approval. The point of contact regarding the availability of data and materials is the corresponding author.

## Abbreviations

CAMHS: Child and Adolescent Mental Health Services
ED: Emergency Department
NHS: National Health Service
NIHR: National Institute for Health and Care Research
NoMAD: Normalisation Measure Development questionnaire
NPT: Normalisation Process Theory
