# Supplementary material for: Towards a typology of mental health crisis care services for children and young people in England and Wales: a cross-sectional survey and analysis of implementation practices
Source: BMC Health Serv Res. 2025 Dec 8;25:1587. doi: 10.1186/s12913-025-13446-6 (PMC12690825; doi:10.1186/s12913-025-13446-6)
Supplement: Supplementary file 1 — Supplementary Material 1 [file 12913_2025_13446_MOESM1_ESM.pdf]

Service characteristics

Please tell us about the service you provide to children and young people who are experiencing extreme psychosocial distress, with or without self-harm, also referred to as a 'crisis'. ***If your service is open to all ages, please respond to this survey in relation to provision for children and young people who are 25 or under***.

1. Your details

Please provide your name and role so that we can contact you for further information if required.

Name	
Role	
Email	

2. Service/project name

Please enter the name of the service or project providing care for children and young people experiencing mental health crisis.

3. Service website

Please enter the website URL for the service (if relevant).

4. Service provider

Please enter the name(s) of the organisation(s) hosting the service, e.g. Greater Manchester Mental Health Trust; Cwm Taf University Health Board; Young Minds.

5. Service commissioner

Who commissions the service (if applicable)? e.g. name of Integrated Care Board, name of Health Board.

6. Service setting

In which setting(s) is the service provided? Please tick all that apply.

- ☐ Accident and emergency department
- ☐ Community health site
- ☐ Community (non-health) site - please specify below:

- ☐ Criminal justice setting
- ☐ Education sector: state-funded mainstream school
- ☐ Education sector: independent school
- ☐ Education sector: special educational needs and disabilities
- ☐ Education sector: sixth form, further education college
- ☐ Education sector: higher education
- ☐ Education sector: school alternative - please specify below:

- ☐ Education/NHS partnership
- ☐ Home setting
- ☐ Inpatient setting
- ☐ Online/virtual
- ☐ Outpatient setting
- ☐ Telephone
- ☐ Youth group
- ☐ Other, please specify:

- ☐ Don’t know

7. Service history

For how long has the service been operating?

- ☐ Under 1 year
- ☐ 1-5 years
- ☐ Over 5 years
- ☐ Don’t know

8. Language provision

In which languages is your service available? Please tick all that apply.

- ☐ English
- ☐ Welsh
- ☐ Other, please specify:

How crisis responses are organised

Please tell us about how the service you provide to children and young people who are experiencing mental health crisis is organised. ***If your service is open to all ages, please respond to these questions in relation to provision for children and young people who are 25 or under***.

9. Focus of service

Is the service a bespoke crisis service that only provides crisis care, or is crisis care part of the overall service specification? Please tick the box that best describes your service.

- ☐ The service is a bespoke crisis service that only provides crisis care
- ☐ Crisis care is part of the overall service specification

10. Defining crisis

Please describe how your service defines ‘mental health crisis’.

11. Criteria for access
Please describe the criteria applied by your service for children and young people to access crisis care.

12. Times of operation
Please specify the operating hours of the service.

- ☐ 7 days a week, 24 hours
- ☐ 7 days a week, extended hours (e.g. 8am - 8pm)
- ☐ 7 days a week, working hours (e.g. 9am - 5pm)
- ☐ Working week (Monday - Friday), 24 hours
- ☐ Working week (Monday - Friday), extended hours (e.g. 8am - 8pm)
- ☐ Working week (Monday - Friday), working hours (e.g. 9am - 5pm)
- ☐ Other, please specify:

- ☐ Don't know

13. Response times
Please indicate the target time within which your service seeks to respond to a request for help in a crisis.

- ☐ Within 2 hours
- ☐ Within 4 hours
- ☐ Within 8 hours
- ☐ Within 24 hours
- ☐ Within 48 hours
- ☐ Other, please specify:

- ☐ Don't know

14. Staffing profile
Please indicate the roles of the people who are involved in providing crisis care for children and young people in your service. Please tick all that apply.

- ☐ Clinical psychologist
- ☐ Assistant psychologist
- ☐ Mental health nurse
- ☐ Children’s nurse
- ☐ Learning disabilities nurse
- ☐ Psychological wellbeing practitioner
- ☐ Psychiatrist
- ☐ Counsellor/therapist
- ☐ Health or social care support worker
- ☐ Occupational therapist
- ☐ Art therapist
- ☐ Drama therapist
- ☐ Health visitor
- ☐ Youth worker
- ☐ Teacher
- ☐ Social worker
- ☐ Peer worker
- ☐ Physiotherapist
- ☐ Volunteer
- ☐ Other, please specify:

Service user characteristics

Please tell us about who accesses your service.

15. Age group served
Is the service a bespoke crisis service for children and young people or is it open to all ages? Please tick the box that best describes your service.

- ☐ The service is a bespoke crisis service for children and young people
- ☐ The service is open to all ages

If your service is open to all ages, please focus on the <u>children and young people</u> who access your service when answering the following questions.

16. Ages of service users

With what age group(s) of children and young people does your service work? Please tick all that apply.

- ☐ < 5 years
- ☐ 5-11 years
- ☐ 12-16 years
- ☐ 17-18 years
- ☐ 19-21 years
- ☐ 22-25 years
- ☐ Other, please specify:

- ☐ Don't know

17. Nature of locality

Which of the following best describes the type of area(s) in which the children and young people you serve live? Please tick all that apply.

- ☐ Rural
- ☐ Semi-rural
- ☐ Town
- ☐ City/metropolitan
- ☐ Online (virtual) service
- ☐ Other, please specify:

- ☐ Don't know

18. Specific groups

Has your service been established with the aim of responding to particular groups of children and young people? Please tick all that apply.

- ☐ No particular group of children and young people is specifically targeted by the service (*please proceed to question 19*)
- ☐ The service is gender-specific (please provide further details):

- ☐ The service is specifically for children and young people from particular ethnic groups (please provide further details):

- ☐ The service is specifically for children and young people of low socio-economic status
- ☐ The service is specifically for children and young people who are refugees/asylum

seekers

- ☐ The service is specifically for children and young people who identify as lesbian, gay, bisexual, transgender, queer/questioning+ (LGBTQ+)
- ☐ The service is specifically for children and young people who are care leavers, looked-after or adopted
- ☐ The service is specifically for children and young people who are disabled
- ☐ The service is specifically for children and young people who are homeless
- ☐ Other, please specify:

- ☐ Don't know

19. Service use

In the last 12 months, approximately how many children and young people have used your service?

- ☐ Fewer than 100
- ☐ Between 101 and 300
- ☐ Between 301 and 500
- ☐ Over 500
- ☐ Don’t know

Service delivery

Please tell us about the services you provide.

20. Goal(s) of crisis intervention

Please tell us what the goal(s) of your service are.

21. Accessing services
How do service users access your service? Please tick all that apply.

☐ Self-referral
☐ Referral via GP or other primary care professional
☐ Referral via educational establishment
☐ Referral via parents/carers
☐ Referral via social care provider
☐ Referral via A&E
☐ Referral via paediatric services
☐ Referral via child and adolescent mental health services
☐ Other, please specify:

☐ Don't know

22. Type of intervention(s)/therapeutic approach(es)
If applicable, please describe the ***type of intervention(s)/therapeutic approach(es)*** you provide, e.g. triage/assessment only, solution-focused brief therapy, multi-systemic therapy, motivational therapy.

23. Delivery of interventions

Which of the following ***modes of delivery*** are employed by your service during core hours? Please tick all that apply.

- ☐ In-person
- ☐ Telephone
- ☐ Online video
- ☐ Text-based/SMS
- ☐ Peer support
- ☐ Web-based online 'live' chat
- ☐ Web-based online forums (e.g. message boards)
- ☐ App-based contact
- ☐ E-mail
- ☐ Other, please specify:

- ☐ Don't know

24. Service users

When providing care in a crisis, with whom does the service directly work? Please tick all that apply.

- ☐ Children and young people
- ☐ Family members or carers
- ☐ Another third party (e.g. professionals such as teachers or social workers, friends, flat mates)

25. Service access

Please indicate whether children and young people's access to your service is restricted in any way. Please tick all that apply.

- ☐ There are no restrictions on access to the service
- ☐ Access is restricted to those who are already using child and adolescent mental health services
- ☐ The service is restricted to a maximum number of sessions
- ☐ The service is restricted to a maximum length of time
- ☐ Other restriction, please specify:

- ☐ Don't know

CAMH-Crisis2
Mapping and evaluating crisis care for children and young people

FUNDED BY NIHR | National Institute for Health and Care Research

26. Frequency of contact with service users
Please specify the average frequency of contact with service users

- ☐ More than once daily
- ☐ Daily
- ☐ Twice weekly
- ☐ Weekly
- ☐ Other, please specify:

[ ]

- ☐ Don't know

Evaluation and research

Please tell us about any previous evaluations or research.

27. Past evaluation or research
If your crisis service for children and young people has been evaluated or researched in the past, please give details below including any information on where this can be found (e.g. website URL, local report, journal article).

[ ]

Implementation of crisis care

Some crisis services for children and young people are very new, while others are well-established. This part of the survey asks questions about ***the implementation of crisis care for children and young people*** in your particular service.

28. From the statements below please tick the option that best describes ***your main role*** in relation to crisis care for children and young people:
    - ☐ I am involved in managing or overseeing crisis care for children and young people
    - ☐ I am involved in delivering crisis care for children and young people

Please answer all the statements below from the perspective of this role. Depending on your role, some statements may be more relevant than others.

Cardiff University / Prifysgol Caerdydd
Manchester 1824 The University of Manchester
University of Plymouth
GIG Cymru NHS Wales Bwrdd Iechyd Prifysgol Aneurin Bevan University Health Board

29. When you provide crisis care for children and young people, how familiar does it feel?

Still feels very new									Feels completely familiar	
0	1	2	3	4	5	6	7	8	9	10

30. Do you feel that providing crisis care for children and young people is currently a normal part of your work?

Not at all					Somewhat					Completely
0	1	2	3	4	5	6	7	8	9	10

31. If you chose numbers 8-10 for question 29, please tick 'not applicable' for this question. Otherwise, please proceed.

☐ Not applicable

Do you feel that providing crisis care for children and young people will become a normal part of your work?

Not at all					Somewhat					Completely
0	1	2	3	4	5	6	7	8	9	10

32. If you have time, please consider each statement below. For each statement, there is the option to agree or disagree with what is being asked **(OPTION A).** However, if you feel that the statement is not relevant to you, there are also options to tell us why **(OPTION B).** Please take the time to decide which answer ***best suits your experience for each statement and tick the appropriate box***.

	Option A					Option B		
	Strongly agree	Agree	Neither agree nor disagree	Disagree	Strongly disagree	Not relevant to my role	Not relevant at this stage	Not relevant to the intervention
I can see how crisis care for children and young people differs from usual ways of working								
Staff in this service have a shared understanding of the purpose of crisis care for children and young people								
I understand how crisis care for children and young people affects the nature of my own work								
I can see the potential value of crisis care for children and young people for my work								

	Strongly agree	Agree	Neither agree nor disagree	Disagree	Strongly disagree	Not relevant to my role	Not relevant at this stage	Not relevant to the intervention
There are key people who drive crisis care for children and young people forward and get others involved								
I believe that participating in crisis care for children and young people is a legitimate part of my role								
I'm open to working with colleagues in new ways to use crisis care for children and young people								
I will continue to support crisis care for children and young people								
I can easily integrate crisis care for children and young people into my existing work								
Crisis care for children and young people disrupts working relationships								
I have confidence in other people's ability to use crisis care for children and young people								
Work is assigned to those with skills appropriate to crisis care for children and young people								
Sufficient training is provided to enable staff to implement crisis care for children and young people								
Sufficient resources are available to support crisis care for children and young people								
Management adequately supports crisis care for children and young people								
I am aware of reports about the effects of crisis care for children and young people								

	Strongly agree	Agree	Neither agree nor disagree	Disagree	Strongly disagree	Not relevant to my role	Not relevant at this stage	Not relevant to the intervention
The staff agree that crisis care for children and young people is worthwhile								
I value the effects that crisis care for children and young people has had on my work								
Feedback about crisis care for children and young people can be used to improve it in the future								
I can modify how I work with crisis care for children and young people								

Questions 28-32: Copyright © Newcastle University 2014. Finch et al., 2013. Improving the normalization of complex interventions: measure development based on normalization process theory (NoMAD): study protocol. Implementation Science 2013, 8:43.
Development of this survey was funded by the Economic and Social Research Council; Study Grant RES-062-23-3274.

Thank you so much for taking the time to complete this survey. Please email to CAMH-Crisis2@cardiff.ac.uk

or post to Dr Leanne Sawle, Room 12.08, School of Healthcare Sciences, Cardiff University, Eastgate House, 35-43 Newport Road, Cardiff, CF24 0AB
