# Supplementary material for: Towards a typology of mental health crisis care services for children and young people in England and Wales: a cross-sectional survey and analysis of implementation practices
Source: BMC Health Serv Res. 2025 Dec 8;25:1587. doi: 10.1186/s12913-025-13446-6 (PMC12690825; doi:10.1186/s12913-025-13446-6)
Supplement: Supplementary file 2 — Supplementary Material 2 [file 12913_2025_13446_MOESM2_ESM.pdf]

CAMH-Crisis2
Mapping and evaluating crisis care for children and young people

FUNDED BY NIHR | National Institute for Health and Care Research

Nodweddion gwasanaeth

Dywedwch wrthym am y gwasanaeth rydych chi'n ei ddarparu i blant a phobl ifanc sy'n profi trallod seicogymdeithasol eithafol, gyda neu heb hunan-niweidio, y cyfeirir ato hefyd fel 'argyfwng'. ***Os yw eich gwasanaeth yn agored i bob oedran, ymatebwch i'r arolwg hwn mewn perthynas â'r ddarpariaeth ar gyfer plant a phobl ifanc sy'n 25 oed neu'n iau***.

1. Eich manylion

Rhowch eich enw a'ch rôl fel y gallwn gysylltu â chi am ragor o wybodaeth os oes angen.

Enw
Rôl
E-bost

2. Enw’r gwasanaeth/prosiect

Nodwch enw’r gwasanaeth neu’r prosiect sy’n darparu gofal I blant a phobl infanc sy’n dioddef o argyfwng iechyd meddwl.

3. Gwefan y gwasanaeth

Rhowch URL gwefan y gwasanaeth (os yw'n berthnasol).

4. Darparwr y gwasanaeth

Nodwch enw(au)’r sefydliad(au) sy’n cynnal y wefan, e.e. Ymddiriedolaeth Iechyd Meddwl Manceinion Fwyaf, Bwrdd Iechyd Prifysgol Cwm Taf, Young Minds.

5. Comisiynydd y gwasanaeth

Pwy sy'n comisiynu'r gwasanaeth (os yw’n berthnasol)? E.e. enw'r Bwrdd Gofal Integredig, enw'r Bwrdd Iechyd.

Cardiff University Prifysgol Caerdydd
Manchester 1824 The University of Manchester
University of Plymouth
GIG Cymru NHS Wales Bwrdd Iechyd Prifysgol Aneurin Bevan University Health Board

6. Lleoliad y gwasanaeth

Ym mha leoliad(au) y darperir y gwasanaeth? Ticiwch bob un sy'n berthnasol.

- ☐ Adran damweiniau ac achosion brys
- ☐ Safle iechyd cymunedol
- ☐ Safle cymunedol (and yw'n ymwneud ag iechyd), rhowch fanylion

- ☐ Lleoliad cyfiawnder troseddol
- ☐ Sector addysg: ysgol prif ffrwd a ariennir gan y wladwriaeth
- ☐ Sector addysg: ysgol annibynnol
- ☐ Sector addysg: anghenion addysgol arbennig ac anableddau
- ☐ Sector addysg: chweched dosbarth, coleg addysg bellach
- ☐ Sector addysg: addysg uwch
- ☐ Sector addysg: ysgol amgen, rhowch fanylion

- ☐ Partneriaeth addysg/GIG
- ☐ Lleoliad cartref
- ☐ Lleoliad cleifion mewnol
- ☐ Ar-lein/rhithwir
- ☐ Lleoliad cleifion allanol
- ☐ Ffôn
- ☐ Grŵp ieuenctid
- ☐ Arall, rhowch fanylion

- ☐ Ddim yn gwybod

7. Hanes y gwasanaeth

Pa mor hir y mae'r gwasanaeth wedi bod yn gweithredu?

- ☐ Llai na blwyddyn
- ☐ 1-5 mlynedd
- ☐ Dros 5 mlynedd
- ☐ Ddim yn gwybod

8. Darpaiaeth iaith

Ym mha ieithoedd y mae eich gwasanaeth ar gael? Ticiwch bob un sy'n berthnasol.

- ☐ Saesneg
- ☐ Cymraeg
- ☐ Arall, rhowch fanylion:

Sut mae ymatebion argyfwng yn cael eu trefnu

Cofiwch sôn wrthym am sut mae'r gwasanaeth rydych chi'n ei ddarparu i blant a phobl ifanc sy'n dioddef o argyfwng iechyd meddwl yn cael ei drefnu. ***Os yw eich gwasanaeth yn agored i bob oedran, ymatebwch i'r arolwg hwn mewn perthynas â'r ddarpariaeth ar gyfer plant a phobl ifanc sy'n 25 oed neu'n iau.***

9. Ffocws y gwasanaeth

A yw'r gwasanaeth yn wasanaeth argyfwng pwrpasol sydd ond yn darparu gofal argyfwng, neu a yw gofal mewn argyfwng yn rhan o fanyleb gyffredinol y gwasanaeth? Ticiwch y blwch sy'n disgrifio eich gwasanaeth orau.

- ☐ Mae'r gwasanaeth yn wasanaeth argyfwng pwrpasol sydd ond yn darparu gofal mewn argyfwng
- ☐ Mae gofal argyfwng yn rhan o fanyleb gyffredinol y gwasanaeth

10. Diffinio argyfwng

Disgrifiwch sut mae eich gwasanaeth yn diffinio 'argyfwng iechyd meddwl'.

11. Meini prawf ar gyfer mynediad
Disgrifiwch y meini prawf a ddefnyddir gan eich gwasanaeth i blant a phobl ifanc gael mynediad i ofal mewn argyfwng.

12. Oriau gweithredu
Nodwch oriau gweithredu'r gwasanaeth

- ☐ 7 diwrnod yr wythnos, 24 awr
- ☐ 7 diwrnod yr wythnos, oriau estynedig (e.e. 8am - 8pm)
- ☐ 7 diwrnod yr wythnos, oriau gwaith (e.e. 9am - 5pm)
- ☐ Wythnos waith (dydd Llun-dydd Gwener), 24 awr
- ☐ Wythnos waith (dydd Llun-Dydd Gwener), oriau estynedig (e.e. 8am - 8pm)
- ☐ Wythnos waith (dydd Llun-dydd Gwener), oriau gwaith (e.e. 9am - 5pm)
- ☐ Arall, rhowch fanylion:
- ☐ Ddim yn gwybod

13. Amserau ymateb
Nodwch yr amser targed i'ch gwasanaeth geisio ymateb i gais am gymorth mewn argyfwng oddi mewn iddo.

- ☐ O fewn 2 awr
- ☐ O fewn 4 awr
- ☐ O fewn 8 awr
- ☐ O fewn 24 awr
- ☐ O fewn 48 awr
- ☐ Arall, rhowch fanylion:

☐ Ddim yn gwybod

14. Proffil staffio
Nodwch rolau'r bobl sy'n ymwneud â darparu gofal mewn argyfwng i blant a phobl ifanc yn eich gwasanaeth. Ticiwch bob un sy'n berthnasol

- ☐ Seicolegydd clinigol
- ☐ Seicolegydd cynorthwyol
- ☐ Nyrs iechyd meddwl
- ☐ Nyrs blant
- ☐ Nyrs anableddau dysgu
- ☐ Ymarerydd lles seicolegol
- ☐ Seiciatrydd
- ☐ Cwnselydd/therapydd
- ☐ Gweithwr cymorth iechyd neu ofal cymdeithasol
- ☐ Therapydd galwedigaethol
- ☐ Therapydd celf
- ☐ Therapydd drama
- ☐ Ymwelydd iechyd
- ☐ Gweithiwr ieuenctid
- ☐ Athro/athrawes
- ☐ Gweithiwr cymdeithasol
- ☐ Gweithiwr cymheiriaid
- ☐ Ffisiotherapydd
- ☐ Gwirfoddoli
- ☐ Arall, rhowch fanylion:

Nodweddion defnyddwyr y gwasanaeth

Dywedwch wrthym am bwy sy'n cyrchu eich gwasanaeth.

15. Grŵp oedran a wasanaethir
A yw'r gwasanaeth yn wasanaeth argyfwng pwrpasol i blant a phobl ifanc neu a yw'n agored i bob oed? Ticiwch y blwch sy'n disgrifio'ch gwasanaeth orau.

- ☐ Mae'r gwasanaeth yn wasanaeth argyfwng pwrpasol i blant a phobl ifanc
- ☐ Mae'r gwasanaeth ar gael i bob oedran

Os yw eich gwasanaeth yn agored i bob oedran, canolbwyntiwch ar y plant a'r bobl

ifanc sy'n cael mynediad i'ch gwasanaeth wrth ateb y cwestiynau canlynol

16. Oedran defnyddwyr y gwasanaeth

Gyda pha grŵp/grwpiau plant a phobl ifanc y mae eich gwasanaeth yn gweithio? Ticiwch bob un sy'n berthnasol.

- ☐ < 5 oed
- ☐ 5-11 oed
- ☐ 12-16 oed
- ☐ 17-18 oed
- ☐ 19-21 oed
- ☐ 22-25 oed
- ☐ Arall, rhowch fanylion:

- ☐ Ddim yn gwybod

17. Natur yr ardal

Pa un o'r canlynol sy'n disgrifio'r math o ardal(oedd) lle mae'r plant a'r bobl ifanc rydych chi'n eu gwasanaethu'n fyw? Ticiwch bob un sy'n berthnasol

- ☐ Gwledig
- ☐ Lled-wledig
- ☐ Tref
- ☐ Dinas/metropolitan
- ☐ Gwasanaeth ar-lein (rhithwir)
- ☐ Arall, rhowch fanylion:

- ☐ Ddim yn gwybod

18. Grwpiau penodol

Ydy eich gwasanaeth wedi’i sefydlu gyda'r nod o ymateb i grwpiau penodol o blant a phobl ifanc? Ticiwch bob un sy'n berthnasol.

- ☐ Does dim un grŵp penodol o blant a phobl ifanc wedi'i dargedu'n benodol gan y gwasanaeth (ewch ymlaen at gwestiwn 19)
- ☐ Mae’r gwasanaeth yn ymwneud â rhywedd penodol (rhowch fanylion):

- ☐ Mae'r gwasanaeth yn benodol ar gyfer plant a phobl ifanc o grwpiau ethnig penodol (rhowch fanylion):

- ☐ Mae'r gwasanaeth yn benodol ar gyfer plant a phobl ifanc sydd â statws economaidd-gymdeithasol isel

- ☐ Mae'r gwasanaeth yn benodol ar gyfer plant a phobl ifanc sy'n ffoaduriaid/ceiswyr lloches
- ☐ Mae'r gwasanaeth yn benodol ar gyfer plant a phobl ifanc sy'n nodi eu bod yn bobl lesbiaidd, hoyw, deurywiol, trawsryweddol, cwiar/sy’n cwestiynu+ (LHDTC+)
- ☐ Mae'r gwasanaeth yn benodol ar gyfer plant a phobl ifanc sy'n gadael gofal, yn derbyn gofal neu'n cael eu mabwysiadu
- ☐ Mae'r gwasanaeth yn benodol ar gyfer plant a phobl ifanc sy'n anabl
- ☐ Mae'r gwasanaeth yn benodol ar gyfer plant a phobl ifanc sy'n ddigartref
- ☐ Arall, rhowch fanylion:

- ☐ Ddim yn gwybod

19. Defnydd o’r gwasanaeth
Yn ystod y 12 mis diwethaf, tua sawl plentyn a pherson ifanc sydd wedi defnyddio eich gwasanaeth?

- ☐ Llai na 100
- ☐ Rhwng 101 and 300
- ☐ Rhwng 301 and 500
- ☐ Mwy na 500
- ☐ Ddim yn gwybod

Darparu gwasanaethau

Dywedwch wrthym am y gwasanaethau rydych yn eu darparu.

20. Nod(au) ymyrraeth argyfwng
Dywedwch wrthym beth yw nod(au) eich gwasanaeth.

21. Cael mynediad i wasanaethau
Sut mae defnyddwyr gwasanaeth yn cael mynediad i'ch gwasanaeth? Ticiwch bob un sy'n berthnasol.

- ☐ Hunanatgyfeirio
- ☐ Atgyfeirio gan feddyg teulu neu weithiwr gofal sylfaenol arall
- ☐ Atgyfeirio gan sefydliad addysgol
- ☐ Atgfyeirio gan rieni/gofalwyr
- ☐ Atgyfeirio gan ddarparwr gofal cymdeithasol
- ☐ Atgyfeirio gan yr Adran Damweiniau ac Achosion Brys
- ☐ Atgyfeirio gan wasanaethau pediatrig
- ☐ Atgyfeirio gan wasanaethau iechyd meddwl plant a phobl ifanc
- ☐ Arall, rhowch fanylion:

- ☐ Ddim yn gwybod

22. Math o ymyrraeth/ymyriadau/dull(iau) therapiwtig
Os yw'n berthnasol, disgrifiwch y ***math o ymyrraeth/ymyriadau/dull(iau) therapiwtig*** a ddarperir gennych, e.e. brysbennu/asesu yn unig, therapi byr sy'n canolbwyntio ar ddatrysiad, therapi aml-systemig, therapi ysgogol.

23. Cyflwyno ymyriadau

Pa rai o'r ***dulliau cyflwyno*** canlynol sy'n cael eu defnyddio gan eich gwasanaeth yn ystod oriau craidd? Ticiwch bob un sy'n berthnasol

- ☐ Wyneb yn wyneb
- ☐ Ffôn
- ☐ Fideo ar-lein
- ☐ Astudiaethau yn seiliedig ar destun/SMS
- ☐ Cefnogaeth gan gymheiriaid
- ☐ Sgwrs 'fyw' ar-lein ar y we
- ☐ Fforymau ar-lein ar y web (e.e. byrddau negeseuon)
- ☐ Cyswllt trwy ap
- ☐ E-bost
- ☐ Arall, rhowch fanylion:

- ☐ Ddim gwybod

24. Defnyddwyr y gwasanaeth

Wrth ddarparu gofal mewn argyfwng, gyda phwy mae'r gwasanaeth yn gweithio'n uniongyrchol? Ticiwch bob un sy'n berthnasol.

- ☐ Plant a phobl ifanc
- ☐ Aelodau teulu neu ofalwyr
- ☐ Trydydd parti arall (e.e. gweithwyr proffesiynol fel athrawon neu weithwyr cymdeithasol, ffrindiau, ffrindiau fflat)

25. Mynediad i'r gwasanaeth

Nodwch a yw mynediad plant a phobl ifanc i'ch gwasanaeth wedi'i gyfyngu mewn unrhyw ffordd. Ticiwch bob un sy'n berthnasol.

- ☐ Nid oes cyfyngiadau ar fynediad i'r gwasanaeth
- ☐ Cyfyngir mynediad i'r rhai sydd eisoes yn defnyddio gwasanaethau iechyd meddwl plant a phobl ifanc
- ☐ Mae'r gwasanaeth wedi'i gyfyngu i uchafswm nifer o sesiynau
- ☐ Mae'r gwasanaeth wedi'i gyfyngu i uchafswm hyd sesiynau
- ☐ Cyfyngiad arall, nodwch:

- ☐ Ddim yn gwybod

26. Pa mor aml y cysylltir â defnyddwyr y gwasanaeth
Nodwch pa mor aml y cysylltir â defnyddwyr y gwasanaeth ar gyfartaledd

- ☐ Mwy nag unwaith bob dydd
- ☐ Bob dydd
- ☐ Ddwywaith yr wythnos
- ☐ Bob wythnos
- ☐ Arall, rhowch fanylion:
- ☐ Ddim yn gwybod

Gwerthuso ac ymchwil

Dywedwch wrthym am unrhyw werthusiadau neu ymchwil blaenorol.

27. Gwerthusiad neu ymchwil blaenorol
A fu unrhyw werthusiad o, neu ymchwil i, eich gwasanaeth argyfwng ar gyfer plant a phobl ifanc? Rhowch fanylion isod, gan gynnwys unrhyw wybodaeth am ble mae hyn i'w weld (e.e. URL y wefan, adroddiad lleol).

Gweithredu gofal argyfwng

Mae rhai gwasanaethau argyfwng i blant a phobl ifanc yn newydd iawn, tra bod eraill wedi hen ennill eu plwyf. Mae'r rhan hon o'r arolwg yn gofyn cwestiynau am ***weithredu gofal mewn argyfwng i blant a phobl ifanc*** yn eich gwasanaeth penodol.

28. O'r datganiadau isod, ticiwch yr opsiwn sy'n disgrifio ***eich prif rôl*** orau mewn perthynas â gofal mewn argyfwng i blant a phobl ifanc:

- ☐ Rwy'n rhan o reoli neu oruchwylio gofal mewn argyfwng i blant a phobl ifanc
- ☐ Rwy'n rhan o'r gwaith o ddarparu gofal mewn argyfwng i blant a phobl ifanc

Atebwch yr holl ddatganiadau isod o safbwynt y rôl hon. Gan ddibynnu ar eich rôl, gall rhai datganiadau fod yn fwy perthnasol nag eraill.

29. Pan fyddwch chi'n darparu gofal mewn argyfwng i blant a phobl ifanc, pa mor gyfarwydd mae'n teimlo?

Dal i deimlo’n newydd iawn										Teimlo’n gwbl gyfarwydd
0	1	2	3	4	5	6	7	8	9	10

30.Ydych chi'n teimlo bod darparu gofal argyfwng i blant a phobl ifanc yn rhan arferol o'ch gwaith ar hyn o bryd?

Ddim o gwbl					I raddau					Yn llwyr
0	1	2	3	4	5	6	7	8	9	10

31. Os dewisoch chi rifau 8-10 ar gyfer cwestiwn 30, ticiwch 'ddim yn berthnasol' ar gyfer y cwestiwn hwn ac ewch ymlaen i gwestiwn 32. Fel arall, ewch ymlaen.

☐ Amherthanasol

Ydych chi'n teimlo y bydd darparu gofal argyfwng i blant a phobl ifanc yn dod yn rhan arferol o'ch gwaith?

Ddim o gwbl					I raddau					Yn llwyr
0	1	2	3	4	5	6	7	8	9	10

32. Os oes gennych amser, ystyriwch bob datganiad isod. Ar gyfer pob datganiad, mae'r opsiwn i gytuno neu anghytuno â'r hyn sy'n cael ei ofyn **(OPSIWN A).** Fodd bynnag, os ydych yn teimlo nad yw'r datganiad yn berthnasol i chi, mae yna opsiynau hefyd i ddweud wrthym pam **(OPSIWN B).** Cymerwch yr amser i benderfynu pa ateb ***sy'n gweddu orau i'ch profiad ar gyfer pob datganiad a thicio'r blwch priodol***.

	Option A					Option B		
	Cytuno’n gryf	Cytuno	Nid cytuno nac yn anghytuno	Anghytuno	Anghytuno’n gryf	Ddim yn berthnasol I fy rôl	Ddim yn berthnasol ar hyn o bryd	Ddim yn berthnasol I’r ymyrraeth
Gallaf weld sut mae gofal mewn argyfwng i blant a phobl ifanc yn wahanol i ffyrdd arferol o weithio								
Mae gan staff yn y gwasanaeth hwn ddealltwriaeth gyffredin o bwrpas gofal mewn argyfwng i blant a phobl ifanc								

	Cytuno’n gryf	Cytuno	Nid cytuno nac yn anghytuno	Anghytuno	Anghytuno’n gryf	Ddim yn berthnasol I fy rôl	Ddim yn berthnasol ar hyn o bryd	Ddim yn berthnasol I’r ymyrraeth
Rwy'n deall sut mae gofal mewn argyfwng i blant a phobl ifanc yn effeithio ar natur fy ngwaith fy hun								
Rwy'n deall sut mae gofal mewn argyfwng i blant a phobl ifanc yn effeithio ar natur fy ngwaith fy hun								
Mae yna bobl allweddol sy'n gyrru gofal mewn argyfwng i blant a phobl ifanc ymlaen a chael eraill i gymryd rhan								
Rwy’n credu bod cymryd rhan mewn gofal mewn argyfwng i blant a phobl ifanc yn rhan ddilys o’m rôl								
Rwy'n agored i weithio gyda chydweithwyr mewn ffyrdd newydd o ddefnyddio gofal mewn argyfwng i blant a phobl ifanc								
Byddaf yn parhau i gefnogi gofal mewn argyfwng i blant a phobl ifanc								
Gallaf integreiddio gofal mewn argyfwng yn hawdd i blant a phobl ifanc i'm gwaith presennol								
Mae gofal mewn argyfwng i blant a phobl ifanc yn amharu ar berthnasoedd Gwaith								

	Cytuno'n gryf	Cytuno	Nid cytuno nac yn anghytuno	Anghytuno	Anghytuno'n gryf	Ddim yn berthnasol I fy rôl	Ddim yn berthnasol ar hyn o bryd	Ddim yn berthnasol I'r ymyrraeth
Mae gen i hyder yng ngallu pobl eraill i ddefnyddio gofal mewn argyfwng i blant a phobl ifanc								
Mae gwaith yn cael ei neilltuo i'r rhai sydd â sgiliau sy'n briodol i ofal mewn argyfwng i blant a phobl ifanc								
Darperir digon o hyfforddiant i alluogi staff i weithredu gofal mewn argyfwng i blant a phobl ifanc								
Mae digon o adnoddau ar gael i gefnogi gofal mewn argyfwng i blant a phobl ifanc								
Mae rheolwyr yn cefnogi gofal mewn argyfwng yn ddigonol i blant a phobl ifanc								
Rwy'n ymwybodol o adroddiadau am effeithiau gofal mewn argyfwng i blant a phobl ifanc								
Mae'r staff yn cytuno bod gofal mewn argyfwng i blant a phobl ifanc yn werth chweil								
Rwy'n gwerthfawrogi'r effeithiau y mae gofal mewn argyfwng i blant a phobl ifanc wedi'i chael ar fy ngwaith								

	Cytuno'n gryf	Cytuno	Nid cytuno nac yn anghytuno	Anghytuno	Anghytuno'n gryf	Ddim yn berthnasol I fy rôl	Ddim yn berthnasol ar hyn o bryd	Ddim yn berthnasol I'r ymyrraeth
Gellir defnyddio adborth am ofal mewn argyfwng i blant a phobl ifanc i'w wella yn y dyfodol								
Gallaf addasu sut rwy'n gweithio gyda gofal mewn argyfwng i blant a phobl ifanc								

Cwestiynau 28-32: Hawlfraint © Prifysgol Newcastle 2014. Finch et al., 2013. Improving the normalization of complex interventions: measure development based on normalization process theory (NoMAD): study protocol Implementation Science 2013, 8:43. Ariannwyd datblygiad arolwg NoMAD gan y Cyngor Ymchwil Economaidd a Chymdeithasol; Grant Astudio RES-062-23-3274.

Diolch am roi o'ch amser i gwblhau'r arolwg hwn. E-bostiwch i CAMH-Crisis2@cardiff.ac.uk

neu postiwch i Dr Leanne Sawle, Room 12.08, School of Healthcare Sciences, Cardiff University, Eastgate House, 35-43 Newport Road, Cardiff, CF24 0AB
